# Adenosine A2A Receptor Mediates Inhibition of Synovitis and Osteoclastogenesis after Electroacupuncture in Rats with Collagen-Induced Arthritis

**DOI:** 10.1155/2019/4617464

**Published:** 2019-03-06

**Authors:** Zhong-heng Du, Chun-wu Zhang, Wen-xia Xie, Yong Chen, Wen-jie Cong, Ze-dong Wang, En-pei Wang, Guang-yu Wu, Tian-shen Ye

**Affiliations:** ^1^Department of Acupuncture, The First Affiliated Hospital of Wenzhou Medical University, Wenzhou, Zhejiang 325000, China; ^2^Department of Traditional Chinese Orthopedics & Traumatology, The First Affiliated Hospital of Wenzhou Medical University, Wenzhou, Zhejiang 325000, China; ^3^Department of Traumatology, The First Affiliated Hospital of Wenzhou Medical University, Wenzhou, Zhejiang 325000, China

## Abstract

**Background:**

This study was to investigate the role of adenosine A2A receptors (A2AR) in inhibiting the effect of electroacupuncture (EA) on osteoclastogenesis in collagen-induced arthritis (CIA).

**Methods:**

Wistar rats were divided into four groups: sham-control group, CIA-control group, CIA-EA group, and CIA-EA-SCH58261 (A2AR antagonist) group. We detected tumor necrosis factor-*α* (TNF-*α*), nuclear transcription factor-*κ*B (NF-*κ*B), receptor activator of NF-*κ*B ligand (RANKL), protein kinase A (PKA), and extracellular regulatory protein kinase 1/2 (ERK1/2) in peripheral blood by ELISA. PKA, ERK1/2, and NF-*κ*B in ankle joints were determined by western blotting. We evaluated the arthritis damage by histological examination and determined the number of osteoclasts by tartrate-resistant acid phosphatase (TRAP) staining.

**Results:**

EA treatment downregulated the expression of TNF-*α*, RANKL, PKA, ERK1/2, and NF-*κ*B in peripheral blood but increased the levels of PKA and ERK1/2 in ankle joints. Importantly, EA treatment reduced bone erosion as evidenced by the histological findings and inhibited osteoclastogenesis as revealed by TRAP staining. All these effects of the EA treatment were reversed by combining EA treatment with the A2AR antagonist SCH58261.

**Conclusion:**

Our data suggest that EA treatment activated A2AR. The effects of the A2AR antagonist SCH58261 suggest that the inhibition of osteoclast formation, the inhibition of TNF-*α*, RANKL, and NF-*κ*B expression, and the increase of ERK1/2 are all dependent on this EA-induced A2AR activation. It is therefore likely that these pathways with clearly defined roles in inflammation and bone erosion are at least partially involved in the mediation of the inhibition of synovitis and osteoclast formation induced by EA.

## 1. Introduction

Rheumatoid arthritis (RA) is a chronic-progressive autoimmune disease. The etiology and pathogenesis of the disease are complex and have not yet been fully elucidated. The pathological features of RA are mainly manifested in chronic inflammation and the formation of new blood vessels (neoangiogenesis). Irreversible destruction of cartilage and bone tissue occurs in the process of disease progression. The prognosis of RA is poor and is usually presented as progressive joint damage, deformity, disability, or even early death. Epidemiological studies indicate that the global prevalence of RA is 0.5% to 1.0% and that it is most typical in women and elderly people [1].

Focal bone erosion is one of the major factors leading to irreversible joint damage and disability by RA. Hofbauer and Heufelder pointed out that the most important factor giving rise to articular cartilage and bone erosion is the bone resorption function of osteoclasts [2]. Osteoclasts, which originate from bone marrow precursor cells, are multinucleated macrophages whose bone absorptive capacity is enhanced in the pathological state of RA and mediated by secretion of hydrochloric acid and associated serine proteases such as cathepsin K and tartrate-resistant acid phosphatase (TRAP) on the surface of bones [3, 4]. The formation, function, and activation of osteoclasts necessitate a complex regulatory network. Proteins such as tumor necrosis factor-*α* (TNF-*α*), interleukin-1 (IL-1), macrophage colony stimulating factor (M-CSF), and receptor activator of NF-*κ*B ligand (RANKL) induce transformation of osteoclast precursors into multinucleated osteoclasts [5]. TNF-*α* plays an important role in the process of osteoclastogenesis. Some reports demonstrate that TNF-*α* activates osteoclastogenesis by regulating the expression of RANKL on the cell membrane of osteoblasts or by binding the type 1 TNF-*α* receptor on the cell membrane of osteoclasts [6–8]. TNF-*α* antagonists can suppress both RANKL expression and osteoclast formation. There are many signaling pathways that regulate the activity of osteoclasts, but the mitogen-activated protein kinase (MAPK) signaling pathway is particularly important [9, 10]. Receptor activator of NF-*κ*B (RANK) and RANKL play pivotal roles in osteoclastogenesis. TNF-*α*, IL-1, and other inflammatory factors affect the process of osteoclast differentiation and maturation [11]. There are many intracellular signaling pathways involved in the regulation of osteoclast formation. The extracellular signal-related kinase (ERK) pathway, one of the MAPK pathways, is a major pathway involved in osteoclast formation. It involves MAPK and extracellular regulatory protein kinase (ERK1/2). Following activation, ERK translocates into the nucleus, followed by a series of biochemical reactions to regulate the nuclear transcription factor-*κ*B (NF-*κ*B) pathway to promote osteoclast formation [12].

Adenosine is an important messenger in the anti-inflammatory process. Under conditions of hypoxia, inflammation, or infection, the extracellular adenosine concentration increases rapidly and plays anti-inflammatory and immunoregulatory roles. Goldman* et al*. found that acupuncture of ST36 (*Zusanli*) acupoint can significantly increase the local concentration of extracellular adenosine [13]. Electroacupuncture (EA) increased the concentration of adenosine by modulating CD73 and ADA expression levels both locally and systemically as we demonstrated in an earlier study [14]. Yim* et al*. found that EA at ST36 significantly reduced IL-6, TNF-*α*, and interferon-*γ* in sera of mice with collagen-induced arthritis (CIA) [15]. EA can reduce the inflammatory process and joint damage. It has been documented that the effects of an electrical stimulus of acupuncture points are more effective than those of nonacupoints and that the effect of an electrical stimulus of nonacupoints is the same as that in the sham-control group [16]. In our previous study, we observed that EA exerted a pronounced effect by inhibiting inflammatory changes and reducing joint damage in a murine CIA model mainly by activating the A2A receptor (A2AR) in inflamed synovial tissue [17]. Based on the results of their animal experiments, Mazzon* et al*. also concluded that A2AR agonists decrease disease progression in experimental CIA mice and suppress expression of TNF-*α* and IL-1*β* [18]. When using A2AR antagonists, this phenomenon can be reversed. Varani* et al*. found that the expression of A2AR in lymphocytes was significantly upregulated in RA patients and that there was a negative correlation between RA disease activity as characterized by the DAS score and A2AR expression [19]. They also demonstrated that both TNF-*α* and IL-1*β* are significantly downregulated after treatment with A2AR agonists. The above phenomenon again could be reversed by A2AR antagonists. Mediero* et al*. confirmed in animal studies that activation of A2AR inhibited the ERK pathway and thus suppressed osteoclast formation [20]. Therefore, we hypothesized that the protective mechanism of EA action in RA may be mediated through the activation of A2AR, through regulating the ERK pathway and inflammation, and thereby by inhibiting the formation of osteoclasts.

## 2. Materials and Methods

### 2.1. Animals

Forty male Wistar rats (4-5 weeks of age, weighing 120 ± 10 g) were used for this study. Rats were purchased from Shanghai Silaike Laboratory Animal Co., Ltd. (Shanghai, China), housed in a controlled environment, and provided with standard rodent chow and water* ad libitum*. Animal care was provided in compliance with regulations on protection of animals used for experimental purposes. Ten rats were randomly assigned to the sham-control group. The other rats were subjected to induction of CIA.

### 2.2. Induction of CIA

Type II collagen extracted from bovine articular cartilage (CII, Chondrex, USA) was dissolved in 0.05M acetic acid (2 mg/mL) at 4°C overnight. Then, the solution was emulsified in an equal volume of complete Freund's adjuvant (CFA, Chondrex, USA). Rats were injected intradermally at the base of the tail with 0.5 mL of the emulsion. On day 21 after the first injection, the rats received a second injection of the same emulsion as described before at the base of the tail close to the previous injection site. A detailed description of our protocol was published previously [14].

### 2.3. Experimental Groups

*Sham-Control Group*. The ten rats in this group were injected intradermally at the base of the tail with 0.5 mL of 0.05M acetic acid instead of the emulsion. These rats were only immobilized, using the bag fixation method as detailed in our last study [17], and did not receive any other treatment or intervention.

*CIA-Control Group*. The CIA rats (n=10) received phosphate-buffered saline (PBS, 5mg/kg) by intraperitoneal injection instead of SCH58261, once daily starting on day 35 until day 49. Immobilization of rats was carried out as previously described.

*CIA-EA Group*. Firstly, PBS (5mg/kg) was injected intraperitoneally and each rat was immobilized with a bag. Thereafter, EA was applied. The rat ST36 and SP6 (*Sanyinjiao*) were determined as described in Chinese Acupuncture and Moxibustion [15, 21]. Sterilized acupuncture needles (0.25 mm × 30 mm; Hua Tuo Acupuncture, China) were inserted into ST36 and SP6. ST36 is located longitudinally three body inches below the knee joint and transversely in the middle of the tibialis anterior muscle, while SP6 is located three body inches above the apex of the medial malleolus, behind the tibia. The depth of ST 36 is approximately 7 mm, while that of SP6 is about 5 mm. Eventually, the CIA rats were electrically stimulated with a continuous rectangular wave current (2Hz) for 30 minutes, once daily starting from day 35 to day 49 after the first immunization (n = 10). The voltage (2.0–2.5mV) was adjusted until local muscle contractions could be seen. EA treatment was administered to the left and right hind legs simultaneously.

*CIA-EA-SCH58261 Group*. The CIA rats were treated with EA immediately after intraperitoneal administration of SCH58261 (5mg/kg). EA was carried out as described above once daily starting on day 35 until day 49 (n = 10). EA and immobilization were carried out as described above.

### 2.4. Histological Examination

After the treatment as described above, rats were sacrificed by exsanguination following an intraperitoneal overdose of pentobarbital sodium. Knees and ankles were fixed in 4% paraformaldehyde solution for 24 hours. The specimens were decalcified in 10% EDTA (pH 8.0, Sigma, USA) for 3-4 weeks and then embedded in paraffin. Paraffin-embedded joint tissues were sectioned and stained with hematoxylin and eosin for histologic assessment. The severity of arthritis in the samples was determined histologically by cumulative assessment of synovial inflammation. Damage due to arthritis (histological damage score) was evaluated and scored by an investigator blinded to the treatment regimen. The following morphological criteria were used: score 0, no damage; score 1, edema; score 2, presence of inflammatory cells; and score 3, bone resorption.

### 2.5. Measurement of the Serum Levels of TNF-*α*, RANKL, PKA, ERK1/2, and NF-*Κ*B Using ELISA

Blood was collected from the rats at the time of sacrifice. Serum was prepared from the blood samples by centrifugation in a constant temperature centrifuge (4°C, 10 min, 3000 r/min). The concentrations of TNF-*α*, RANKL, PKA, ERK1/2, and NF-*Κ*B were measured using a commercial ELISA kit according to the manufacturer's instructions (R&D Systems, Minneapolis, MN). The values were normalized for total protein concentration.

### 2.6. Western Blotting

For western blot analysis of PKA, ERK1/2, and NF-*Κ*B, tissue samples were collected from the ankle joints. The specimens excluding bone were cut into small pieces and lysed with RIPA buffer (1% Triton X-100, 1% deoxycholate, 0.1% SDS) containing the protease inhibitor PMSF to extract total protein. The homogenizer contributes to lysis on ice. After lysis for 20 min on ice, the lysates were centrifuged at 10,000 g for 20 min at 4°C. Protein concentration was determined by the BCA method. Samples were subjected to sodium dodecyl sulfate-polyacrylamide gel electrophoresis (SDS-PAGE) on 10% PA gels and electrotransferred onto a nitrocellulose membrane. To block nonspecific binding, membranes were washed with Tris-buffered saline containing 0.05% Tween-20 (TBST, pH 7.6) and then with 5% skim milk at room temperature for 2h. The membranes were incubated overnight at 4°C with the primary antibody (1:5000 dilution for anti-PKA, 1:10000 dilution for anti-ERK1/2, and 1:1000 dilution for anti-NF-*Κ*B). After washing with TBST, the membranes were incubated with horseradish peroxidase- (HRP-) conjugated secondary antibody (1:5000) in blocking buffer at room temperature for 1h. Next, the membranes were washed again with TBST and the chemiluminescence was determined and recorded. Intensities of the respective bands were examined by densitometric analysis using the ImageJ software. To quantify results of western blot analysis, digital densitometric band analysis was performed and band intensities were expressed relative to that of the sham-control group.

### 2.7. TRAP Staining

Osteoclasts were evaluated by staining for TRAP using the Acid Phosphatase Leukocyte TRAP kit (Sigma) according to the manufacturer's protocol. TRAP-positive multinucleated cells containing three or more nuclei were counted as osteoclasts.

### 2.8. Statistical Methods

All data were processed with the SPSS 19.0 software. The data were presented as mean ± standard deviation (SD) and analyzed by one-way ANOVA. Tukey HSD was applied when the homogeneity of variances was uniform; if it was not, Dunnett's T3 test was used for post hoc multiple comparisons. Categorical variables were tested using the nonparametric rank-sum test. Differences with a p value equal to or less than 0.05 were considered statistically significant.

## 3. Results

### 3.1. Histological Evaluation

**Figure 1:**
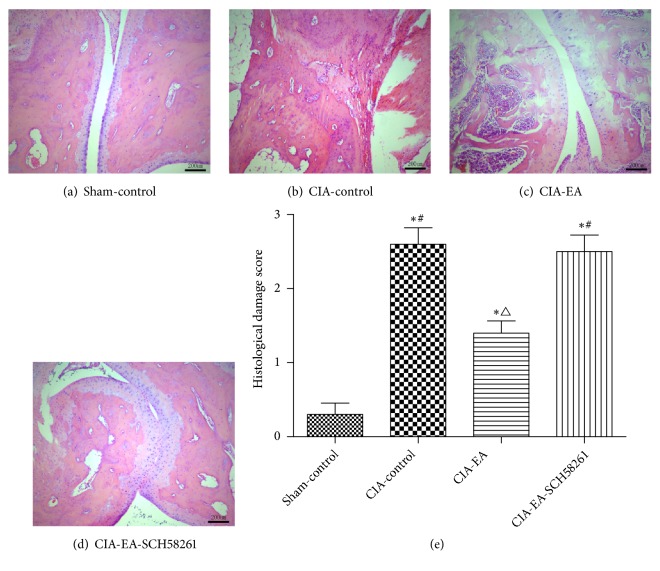
Histological evaluation of the knee in CIA. Hematoxylin-eosin staining of the knee was performed to investigate joint damage. The sham-control group showed the normal structure (a, e). Joint damage was most serious in the CIA-control group, characterized by severe adhesions and infiltration by inflammatory cells, bone erosion, and cartilage damage (b, e). Inflammatory changes and bone erosion were also detectable in the CIA-EA group, but the joint was damaged to a significantly lesser extent compared with the CIA-control group (b, c, and e). Adhesions and bone damage were worse in the CIA-EA-SCH58261 group compared to the CIA-EA group. The CIA-EA-SCH58261 was similar to the CIA-control group (b, c, d, and e). Data were expressed as mean ± SD and were compared using one-way ANOVA and Tukey HSD multiple comparison post hoc test. *∗* indicates P<0.01* versus* the sham-control group. △ indicates P<0.01* versus* the CIA-control group. # indicates P<0.05* versus* the CIA-EA group.

Rats in the sham-control group showed normal knee joint structure. Bone erosion and inflammatory cell infiltration were most serious in the CIA-control group. The joint space disappeared, and bones underwent fusion. The degree of bone erosion and inflammatory cell infiltration in the CIA-EA group was less severe than that observed in the CIA-control group. The histological damage scores were lower in this group. When SCH58261 was administered concurrently with EA, results indicated that the effect of EA in the CIA-EA-SCH58261 group was reversed (Figure 1 and Table 1).

### 3.2. ELLSA Results of Serum TNF-*α* and RANKL Concentrations in Rats

**Figure 2:**
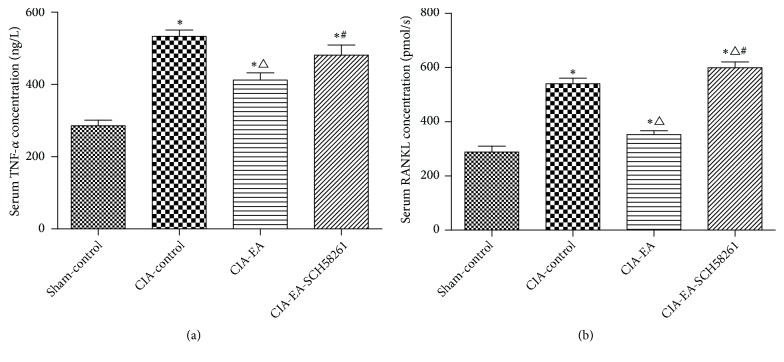
TNF-*α* and RANKL concentrations in different treatment groups. TNF-*α* and RANKL concentrations in serum samples of rats from the CIA-control group were significantly elevated compared to those of the sham-control group. At the same time, we found that EA intervention significantly decreased levels of serum TNF-*α* and RANKL compared with the CIA-control group. When the antagonist SCH58261 and EA were administered simultaneously, the levels of TNF-*α* and RANKL increased significantly in comparison to the CIA-EA group. Data were expressed as mean ± SD and were compared using one-way ANOVA and Tukey HSD multiple comparison post hoc test. *∗* P<0.05* versus* the sham-control group l. △ P<0.05* versus* the CIA-control group. # P<0.05* versus* the CIA-EA group.

The levels of TNF-*α* and RANKL were low in the sham-control group. The expression of TNF-*α* and RANKL was increased significantly in the CIA-control group compared to the sham-control group. The levels of TNF-*α* and RANKL were decreased significantly in the CIA-EA group compared to the CIA-control group. When SCH58261was administered simultaneously with EA, the effect of EA was reversed and the resulting TNF-*α* and RANKL concentrations were similar to those in the CIA-control group (Figure 2 and Tables 2 and 3).

### 3.3. PKA, ERK1/2, and NF-*Κ*B Concentrations in Serum

**Figure 3:**
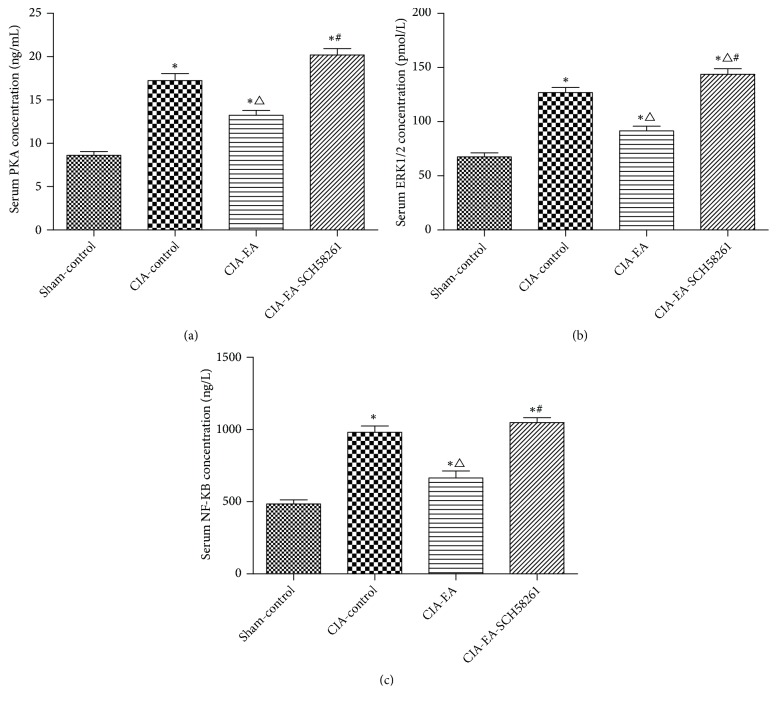
Serum concentrations of PKA, ERK1/2, and NF-*κ*B in different treatment groups. After CIA was established, the concentrations of PKA, ERK1/2, and NF-*κ*B in serum were drastically elevated compared to the sham-control group. EA intervention significantly reduced PKA, ERK1/2, and NF-*κ*B concentrations compared with the CIA-control group. The levels of PKA, ERK1/2, and NF-*κ*B in the CIA-EA-SCH58261 group were higher than those in the CIA-EA group. Data were expressed as mean ± SD and were compared using one-way ANOVA. The PKA data were analyzed by Dunnett's T3 multiple comparison post hoc test. The data of ERK1/2 and NF-*κ*B were analyzed using Tukey HSD. *∗* P<0.01* versus* the Sham-control group. △ indicates P<0.05* versus* the CIA-control group. # indicates P<0.01* versus* the CIA-EA group.

The serum concentrations of PKA, ERK1/2, and NF-*Κ*B, as determined by ELISA, were similar. The expressions of PKA, ERK1/2, and NF-*Κ*B were higher in the CIA-control group than in the sham-control group. When CIA rats received EA treatment, the levels decreased compared to the CIA-control group. The effect of EA treatment was reversed when SCH58261 was coadministered (Figure 3 and Tables 4–6).

### 3.4. Western Blot Analysis of PKA, ERK1/2, and NF-KB Protein in Ankle Joints

**Figure 4:**
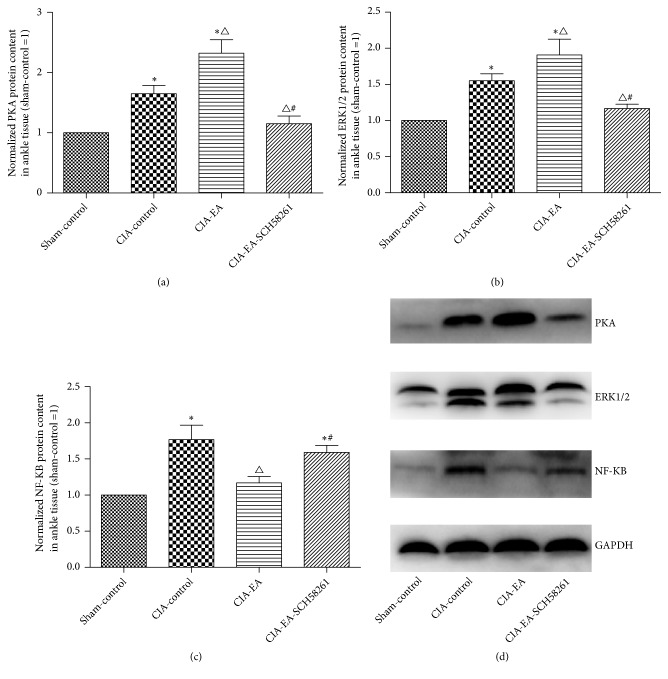
The levels of PKA, ERK1/2, and NF-*κ*B in ankle joints. PKA, ERK1/2, and NF-*κ*B protein content in local ankle joints in the CIA-control group increased compared to the sham-control group. When the rats received EA treatment, the levels of PKA and ERK1/2 increased further compared to the CIA-control group, which is the opposite of the effect observed in serum. The levels of PKA and ERK1/2 decreased significantly compared to the CIA-EA group when the A2AR antagonist was administered simultaneously with the EA treatment. In contrast to PKA and ERK1/2, the local concentration of NF-*κ*B was similar to that in serum. Data were expressed as mean ± SD and were compared using one-way ANOVA and Dunnett's T3 multiple comparison post hoc test. *∗* indicates P<0.01* versus* the sham-control group. △indicates P<0.05* versus* the CIA-control group. # indicates P<0.05* versus* the CIA-EA group.

After CIA was successfully established in rats, PKA, ERK1/2, and NF-*Κ*B were higher in the CIA-control group than in the sham-control group. Additional EA treatment increased PKA and ERK1/2 in comparison to the CIA-control group. The protein concentrations of PKA and ERK1/2 were significantly lower compared to the CIA-EA group when SCH58261 was coadministered with EA. The amount of NF-*Κ*B in ankle joint tissue was similar to that in serum (Figure 4 and Tables 7–9).

### 3.5. Presence of Osteoclasts in Ankle Joint Tissue

**Figure 5:**
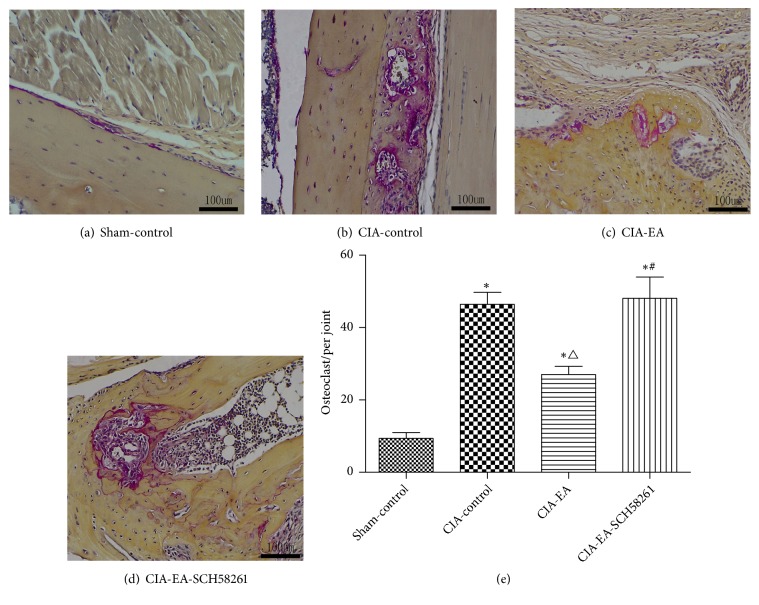
Quantification of osteoclasts per joint. The number of osteoclasts was low in the sham-control group (a, e). In CIA rats, the number of osteoclasts was drastically elevated compared with the sham-control group (a, b, and e). When EA was administered to CIA rats, the count of osteoclasts was lower in comparison to the CIA-control group (b, c, and e). When EA and A2AR antagonist SCH58261 were administered simultaneously, the number of osteoclasts was significantly increased compared to the CIA-EA group (c, d, and e). Osteoclasts were counted per joint. Data were expressed as mean ± SD and were compared using one-way ANOVA and Dunnett's T3 multiple comparison post hoc test. *∗* indicates P<0.01 versus the sham-control group. △ indicates P<0.01 versus the CIA-control group. # indicates P<0.05 versus the CIA-EA group.

In the sham-control group, few osteoclasts were identified by microscopic observation. The number of osteoclasts was significantly higher in the CIA-control group compared to the sham-control group. The osteoclast count was lower in the CIA-EA group compared to the CIA-control group. This effect of EA was reversed when SCH58261 was coadministered (Figure 5 and Table 10).

## 4. Discussion

The adenosine receptors include A1R, A2AR, A2BR, and A3R. A1R is a proinflammatory mediator, while A2AR is an anti-inflammatory mediator. In a previous study, we reported that EA prevented arthritis from causing bone erosion by activing A2AR and by inhibiting TNF-*α* [14, 17]. Elevated A2AR decreased the expression of inflammatory cytokines. TNF-*α* is the most important proinflammatory cytokine* in vivo*. TNF-*α* overexpression leads to the release of a series of other inflammatory factors downstream in the inflammatory pathway, resulting in damage to various tissues. In RA, a condition associated with chronic inflammation, TNF-*α* is continually overexpressed. Some scholars pointed out that TNF-*α* regulates RANKL to activate osteoclastogenesis [8, 22]. RANKL is found in the membrane of osteoblasts, whereas RANK is located in the membrane of osteoclast precursors. When TNF-*α* levels are high, RANKL binding to RANK is stimulated. This initiates the differentiation of osteoclast precursors into mature osteoclasts. The results of our current study indicate that EA treatment inhibits osteoclast formation by suppressing the expression of TNF-*α* and RANKL. In our previous study, we found that EA treatment activated A2AR [17]. When the A2AR was blocked, the TNF-*α* level was upregulated. In our current study, both TNF-*α* and RANKL levels increased significantly when EA treatment and the A2AR antagonist SCH58261 were administered simultaneously. Therefore, we suggest that EA-induced activation of A2AR inhibited osteoclast formation* via* a pathway mediated by TNF-*α* and RANKL.

Osteoclasts play a crucial role in bone erosion in the joint. The main function of osteoclasts is bone resorption. Bone resorptive capacity is enhanced in a pathological state by secretion of hydrochloric acid and associated serine proteases on the surface of bones. Elevated osteoclast activity reduces the bone volume and degrades bone to the primary bone state [20]. Some scholars demonstrated that A2AR inhibits both osteoclast formation and function [20, 23, 24]. The MAPK signaling pathway is also important in A2AR regulation of osteoclasts. The MAPKs are involved in three pathways: the p38, JNK, and ERK1/2 pathway. Mediero* et al*. suggested that only ERK1/2 are associated with A2AR regulation [12]. Activation of A2AR inhibits osteoclast differentiation* via* activation of PKA and ERK1/2. Our results are consistent with the hypothesis of Mediero [12]. EA treatment augments the level of A2AR and thereby upregulates the expression of PKA and ERK1/2 [13–15, 17]. When we used the A2AR antagonist SCH58261 together with EA, PKA and ERK1/2 were downregulated compared to EA alone. Surprisingly, the effects of EA and EA plus SCH58261 on PKA and ERK1/2 in serum were different from the local effects of the same treatment. There are two likely explanations for this phenomenon. Firstly, RA is predominantly a disease of local joint inflammation and therefore the local inflammation in the joint is more severe than the systemic inflammation reflected in peripheral blood. Secondly, EA treatment exerts mainly a local anti-inflammatory effect* via* activating A2AR in the synovial tissue of rats with CIA [17]. Adenosine has a short half-life* in vivo*, and it is quickly hydrolyzed to inosine. Therefore, the local activation of A2AR has no significant systemic effect on kinase levels as represented by the serum levels.

We also investigated the final step in the process of osteoclastogenesis. NF-*κ*B activation is essential for the formation and maturation of osteoclasts. TNF-*α* stimulates RANKL binding to RANK, which leads to NF-*κ*B mediation of the process of osteoclastogenesis. At the end of the ERK1/2 signaling pathway, elevated PKA and ERK1/2 inhibit NF-*κ*B activation and thereby also osteoclastogenesis. Lack of NF-*κ*B activation will prevent osteoclast formation and there will be no TRAP-positive osteoclasts [25]. Our experimental results suggest that EA treatment affects NF-*κ*B activation, thereby inhibiting osteoclast formation.

In summary, we hypothesize that EA treatment can inhibit inflammatory changes and osteoclastogenesis by activating A2AR. EA treatment may have effects on the TNF-*α*, RANKL, and the ERK1/2 pathway. NF-*κ*B is the common final target for both TNF-*α* and ERK1/2. However, there are additional pathways that mediate osteoclastogenesis. Further investigations will elucidate which pathways are the most important ones in mediating the effects of EA treatment.

## 5. Conclusions and Outlook

**Figure 6:**
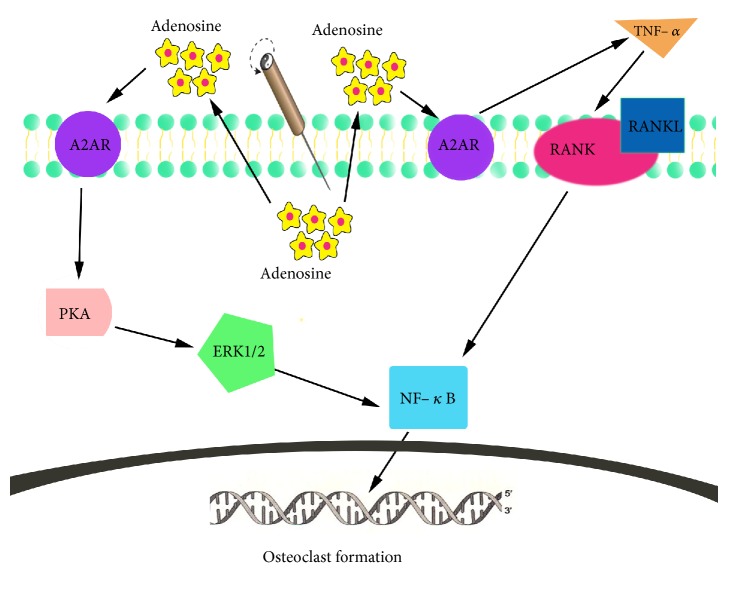
EA ameliorates osteoclastogenesis by inhibiting NF-*κ*B. EA treatment stimulates adenosine release to the extracellular environment, resulting in activation of A2AR. Activated A2AR inhibits TNF-*α* resulting in decreased binding of RANKL to RANK and finally inhibits osteoclast formation by reducing NF-*κ*B activation. Activated A2AR also affects both the PKA and ERK1/2 signaling pathway and thereby inhibits osteoclast formation by reducing expression of NF-*κ*B.

Our study suggests that EA treatment activates A2AR and inhibits the formation of osteoclasts and the expression of TNF-*α*, RANKL, and NF-*κ*B, while it increases the expression of ERK1/2. The effects of the A2AR antagonist SCH58261 indicate that the inhibition of osteoclast formation, the inhibition of TNF-*α*, RANKL, and NF-*κ*B expression, and the increase of ERK1/2 are all dependent on this EA-induced A2AR activation. It is therefore likely that these pathways with clearly defined roles in inflammation and bone erosion are at least partially involved in the mediation of the inhibition of synovitis and osteoclast formation induced by EA (Figure 6). In the future, we will use an A2AR gene knockout animal model to study which additional pathways might be involved in the mediation of the effects of EA.

## Figures and Tables

**Table 1:** Histological damage scores.

	n	Histological damage score
Sham-control group	10	0.30±0.48
CIA-control group	10	2.60±0.70 *∗*
CIA-EA group	10	1.40±0.52 *∗*△
CIA-EA-SCH58261 group	10	2.50±0.71 *∗*#

*∗P*<0.01 *versus* the Sham-control group. △*P*<0.01 *versus* the CIA-control group. # *P*<0.05 *versus* the CIA-EA group.

**Table 2:** Serum TNF-*α* concentration (ng/L).

	n	TNF-*α*
Sham-control group	10	285.93±48.83
CIA-control group	10	533.25±55.78 *∗*
CIA-EA group	10	412.14±63.23 *∗* △
CIA-EA-SCH58261 group	10	481.50±87.36 *∗* #

*∗P*<0.05 *versus* the Sham-control group. △*P*<0.05 *versus* the CIA-control group. # *P*<0.05 *versus* the CIA-EA group.

**Table 3:** Serum RANKL concentration (pmol/L).

	n	RANKL
Sham-control group	10	288.59±69.63
CIA-control group	10	540.64±65.17 *∗*
CIA-EA group	10	352.80±44.15 *∗* △
CIA-EA-SCH58261 group	10	599.72±67.92 *∗* △ #

*∗P*<0.05 *versus* the Sham-control group. △*P*<0.05 *versus* the CIA-control group. # *P*<0.05 *versus* the CIA-EA group.

**Table 4:** Serum PKA concentration (ng/mL).

	n	PKA
Sham-control group	10	8.61±1.37
CIA-control group	10	17.25±2.54 *∗*
CIA-EA group	10	13.24±1.76 *∗* △
CIA-EA-SCH58261 group	10	20.20±2.35 *∗* #

*∗* indicates *P*<0.01 *versus* the Sham-control group. △ indicates* P*<0.05 *versus* the CIA-control group. # indicates* P*<0.01 *versus* the CIA-EA group.

**Table 5:** Serum ERK1/2 concentration (pmol/L).

	n	ERK1/2
Sham-control group	10	67.63±11.26
CIA-control group	10	126.98±14.81 *∗*
CIA-EA group	10	91.53±13.91 *∗* △
CIA-EA-SCH58261 group	10	143.69±16.41 *∗* △ #

*∗* indicates* P*<0.01 *versus* the Sham-control group. △ indicates* P*<0.05 *versus* the CIA-control group. # indicates* P*<0.01 *versus* the CIA-EA group.

**Table 6:** Serum NF-*κ*B concentration (ng/L).

	n	NF-*κ*B
Sham-control group	10	483.96±91.86
CIA-control group	10	981.11±135.02 *∗*
CIA-EA group	10	664.49±152.67 *∗* △
CIA-EA-SCH58261 group	10	1048.41±105.38 *∗* #

*∗* indicates* P*<0.01 *versus* the Sham-control group. △ indicates* P*<0.05 *versus* the CIA-control group. # indicates *P*<0.01 *versus* the CIA-EA group.

**Table 7:** Normalized PKA protein content in ankle tissue (sham-control =1).

	n	PKA
Sham-control group	10	1.00±0.00
CIA-control group	10	1.65±0.43 *∗*
CIA-EA group	10	2.32±0.71*∗*△
CIA-EA-SCH58261 group	10	1.15±0.40△ #

*∗* indicates* P*<0.01 *versus* the Sham-control group. △ indicates* P*<0.05 *versus* the CIA-control group. # indicates* P*<0.05 *versus* the CIA-EA group.

**Table 8:** Normalized ERK1/2 protein content in ankle tissue (sham-control =1).

	n	ERK1/2
Sham-control group	10	1.00±0.00
CIA-control group	10	1.55±0.30 *∗*
CIA-EA group	10	1.91±0.69*∗*△
CIA-EA-SCH58261 group	10	1.17±0.20 △#

*∗* indicates *P*<0.01 *versus* the sham-control. △ indicates* P*<0.05 *versus* the CIA-control group. # indicates* P*<0.05 *versus* the CIA-EA group.

**Table 9:** Normalized NF-*κ*B protein content in ankle tissue (sham-control =1).

	n	NF-*κ*B
Sham-control group	10	1.00±0.00
CIA-control group	10	1.77±0.64 *∗*
CIA-EA group	10	1.17±0.28 △
CIA-EA-SCH58261 group	10	1.59±0.31 *∗* #

*∗* indicates* P*<0.01 *versus* the sham-control group. △ indicates* P*<0.05 *versus* the CIA-control group. # indicates* P*<0.05 *versus* the CIA-EA group.

**Table 10:** Presence of osteoclasts in ankle tissue.

Group	n	Osteoclasts
Sham-control group	10	9.40±4.95
CIA-control group	10	46.40±10.49 *∗*
CIA-EA group	10	27.00±7.18 *∗* △
CIA-EA-SCH58261 group	10	48.10±18.62 *∗* #

TRAP-positive multinucleated cells containing three or more nuclei were counted as osteoclasts. Osteoclasts numbers represent the total numbers in the joint.

*∗* indicates* P*<0.01 *versus* the Sham-control group. △ indicates* P*<0.01 *versus* the CIA-control group. # indicates* P*<0.05 *versus* the CIA-EA group.

## Data Availability

The data used to support the findings of this study are included within the article.
